# Effects of common lifestyle factors on obstructive sleep apnea: precautions in daily life based on causal inferences

**DOI:** 10.3389/fpubh.2024.1264082

**Published:** 2024-03-05

**Authors:** Kun Liu, Chenyang Zang, Jixu Wang, Jie Liu, Ziliang Chen, Meng He, Bin Liu, Xiaoli Su, Yuan Zhang, Minhan Yi

**Affiliations:** ^1^Department of Respiratory Medicine, Xiangya Hospital, Central South University, Changsha, China; ^2^School of Life Sciences, Central South University, Changsha, China; ^3^Department of Medical Imaging Laboratory and Rehabilitation, Xiangnan University, Chenzhou, China; ^4^National Clinical Research Center for Geriatric Disorders, Xiangya Hospital, Central South University, Changsha, China; ^5^Xiangya Medical School, Central South University, Changsha, China

**Keywords:** OSA, lifestyle factor, smoking behavior, alcohol intake, coffee intake

## Abstract

**Background:**

This study aimed to evaluate the causal impact of common modifiable lifestyles on obstructive sleep apnea (OSA), which is beneficial for recommendations to prevent and manage OSA.

**Method:**

Published genome-wide association study (GWAS) summary statistics were used to perform two-sample Mendelian randomization (MR). Variants associated with each exposure of smoking, drinking, and leisure sedentary behaviors at the genetic level were used as instrumental variables (IVs). Then, inverse-variance weighting (IVW) was considered the primary result for causality. Moreover, several complimented approaches were also included to verify the observed associations. MR-PRESSO and MR-Egger intercept were applied to test the horizontal pleiotropy. To assess heterogeneity, Cochran's *Q* test by IVW and MR-Egger were applied.

**Results:**

Regular smoking history increased OSA risk in all applied approaches [OR (95% CI)_IVW_ = 1.28 (1.12, 1.45), *p* = 1.853 × 10^−4^], while the causality of lifetime smoking index [OR (95% CI)_IVW_ = 1.39 (1.00, 1.91), *p* = 0.048], alcohol intake frequency [outliers removed OR (95% CI)_IVW_ = 1.26 (1.08, 1.45), *p* = 0.002], and coffee intake behavior [OR (95% CI)_IVW_ = 1.66 (1.03, 2.68), *p* = 0.039] on OSA risk were not always consistent in other approaches. In addition, no robust causal associations were observed for the effect of sedentary leisure behaviors on OSA risk. In sensitivity analysis, we observed no sign of horizontal pleiotropy or heterogeneity.

**Conclusion:**

Ever regularly smoking has a robust causal role in increasing OSA risk, which should be discouraged as precautions from developing OSA.

## 1 Introduction

Obstructive sleep apnea (OSA) is a common sleep-disordered breathing (SDB) affecting as much as 6%−13% of the common population (1). It is caused by repeated upper airway collapse during sleep, resulting in intermittent hypoxia, and manifests as sleep fragmentation, snoring, and daytime sleepiness. Current common OSA therapies, e.g., positive airway pressure and surgery (2), could relieve sleep apnea symptoms. However, these therapies also come with poor compliance (3) and financial burdens, compromising the quality of life for OSA patients. Lifestyle modifications (4), e.g., weight loss and smoking cessation, have been recommended for OSA patients. However, there is still a gap in offering scientific, evidence-based health advice for which kind of lifestyle is beneficial for the prevention and clinical management of OSA.

Previous clinical research has linked several common lifestyles to OSA pathogenesis. Smoking has been linked to a direct contribution to upper airway inflammation, a factor known to heighten the susceptibility to episodes of sleep apnea (5). Alcohol relaxes muscles, including the muscles in the throat, which can lead to increased airway collapse during sleep (6, 7). This relaxation contributes to the development or worsening of OSA. While stimulant drinks are often associated with wakefulness, they can also disrupt sleep patterns and exacerbate OSA symptoms. Multiple observational studies have described a positive association between cigarette smoking and an increased risk of OSA. On the other hand, alcohol consumption was reported as an independent risk factor for OSA (8), while OSA may increase the incidence of alcohol use disorder in turn (9). Furthermore, smoking, alcohol consumption, and OSA are linked to cardiovascular impairments, several other sleep traits, and morbidity (10–13). Common stimulant drinks such as coffee and tea (14) were also linked to OSA risks, while there is only a limited number of observational studies. Furthermore, leisurely sedentary behaviors such as TV watching, computer use, driving, etc., have been linked to health concerns (15). OSA has been widely reported to negatively affect driving performance through cognitive impairment (16).

Most of the previous observational studies are prone to biases from reverse causation and residual confounding. There is also a possible inverse association that OSA patients may be predisposed to smoking to cope with daytime sleepiness (17) or mental health comorbidities (18). Meanwhile, there are practical difficulties in conducting randomized controlled trials (RCTs) for any of these common lifestyle risk factors, or more specifically taking behavioral details into consideration, e.g., smoking initiation or cessation, and frequency and duration of drinking. For example, chronic smoking over an extended period is more likely to increase the frequency and severity of OSA episodes (19). Adding more to it, heterogeneity in current clinical evidence, selective bias, and potential inverse association may cloud the understanding of the causal relationship between these common lifestyles and OSA. Mendelian randomization (MR) estimates the potential causal relationship between exposure and outcome by using genetic variants as instrumental variables (IV) for the exposure factor of interest. As genetic variants are assigned at random during conception, the MR approach is not susceptible to confounding factors. It can also avoid reverse causality bias, with genetic variants being assigned prior to disease development. Similar methods were applied to study the associations among sleep traits, inflammation protein levels, and glycemic traits (20–22).

Lifestyle factors, such as smoking, alcohol, stimulant drinks, and sedentary behaviors, are implicated in obstructive sleep apnea (OSA) pathogenesis. Utilizing an MR can overcome biases in observational studies, offering a more robust understanding of their causal relationships. In this study, we aimed to evaluate a possible causal association between common modifiable lifestyles (i.e., smoking, alcohol, coffee and tea consumption, TV watching, computer use, and driving) and OSA via an MR approach. Our findings will offer valuable insights for understanding OSA pathogenesis and clinical management of OSA.

## 2 Materials and methods

### 2.1 Overall study design

Generally, we conducted a bidirectional two-sample MR using openly available GWAS data to analyze the causal effects of common modifiable lifestyles on OSA. In the context of our research question, a two-sample MR involves utilizing genetic instruments derived from one dataset to estimate causal effects on the outcome, OSA, observed in a separate dataset. Specifically, instrumental variables associated with modifiable lifestyle factors are used as proxies for exposures. The genetic variants' impact on the exposure is then evaluated in relation to the outcome, ensuring a robust causal inference. Based on three basic assumptions of MR analysis (23):

- Relevance (instrumental variable): the genetic variants used as instrumental variables should be associated with the modifiable exposure of interest (e.g., lifestyle factor) that is being studied.
- Exclusion (no confounding): the selected genetic variants should not have direct associations with any confounding factors that might influence both the exposure and the outcome. This assumption helps ensure that the genetic variants act primarily through the exposure of interest.
- Independence (no pleiotropy): the genetic variants used in the analysis should influence the outcome (e.g., disease) only through the modifiable exposure being studied and not through alternative pathways (pleiotropy).

**Figure 1:**
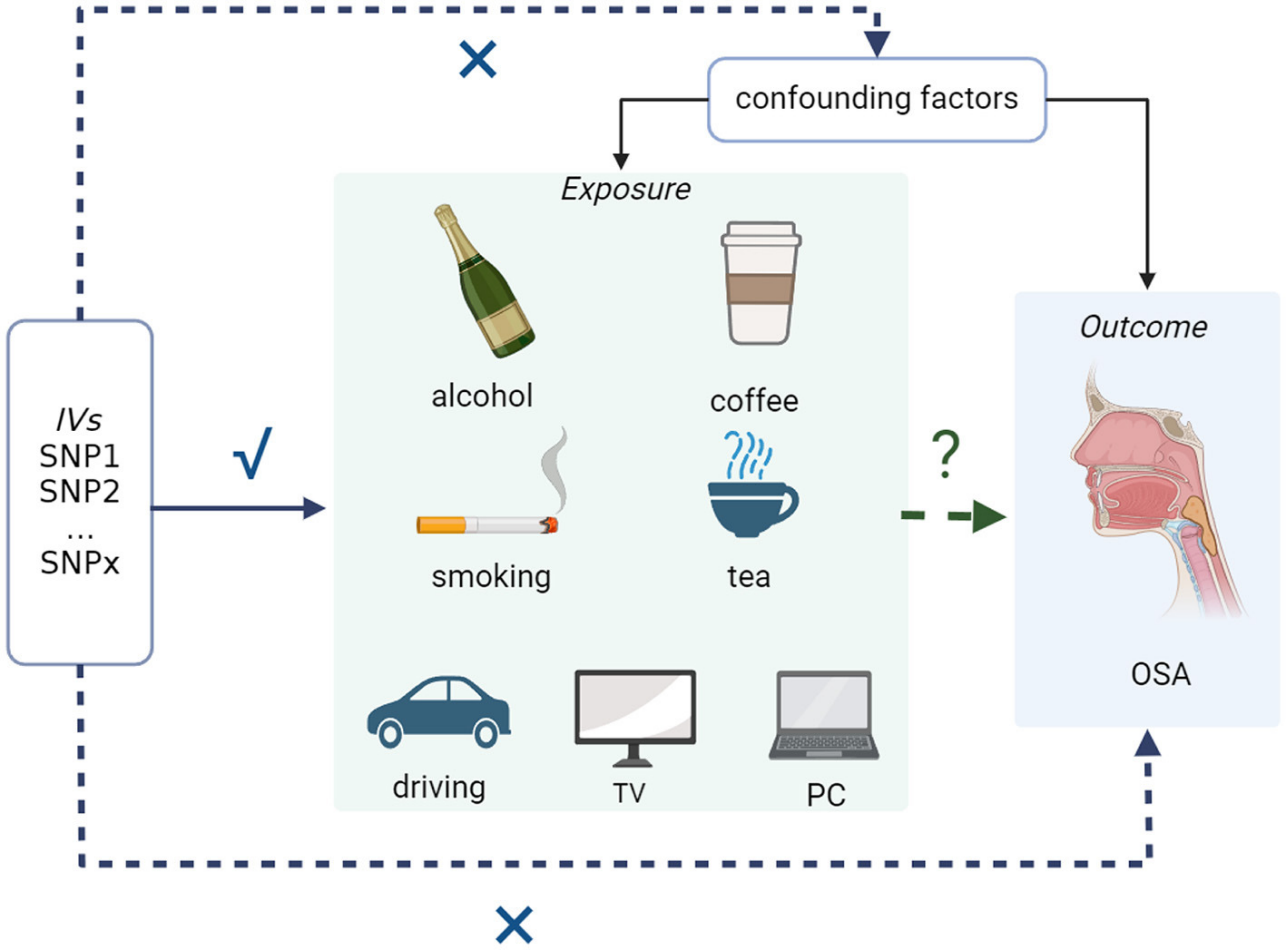
Overall working flowchart: two-sample MR study to assess the causal associations between common modifiable lifestyles and OSA. OSA, obstructive sleep apnea.

First, the genetic variants selected as instrumental variables involved in this study were highly related to exposure. Second, the selected genetic variants had no associations with any confounders. Third, modifiable lifestyles, such as exposure of interest, are the only way through which genetic variants affect OSA. The detailed design and the behaviors included are shown in Figure 1.

### 2.2 Data source

For exposure of interest, the GWAS for smoking were from GSCAN and UK Biobank (24, 25). More specifically, ever smoked regularly was defined as those who had a history of regular smoking, comprised of 1,232,091 participants. Age at initiation of regular smoking was defined as the age at which an individual started smoking cigarettes regularly (*N* = 341,427). Cigarettes per day were defined as the average number of cigarettes smoked per day, as either a current smoker or a former smoker (*N* = 337,334). Smoking cessation was defined as current smokers vs. former smokers and comprised 547,219 participants. The mean age at the time of assessment was 29.0 years (SD = 1.8 years). The lifetime smoking index is a comprehensive measurement of the heaviness of smoking, comprised of smoking initiation, heaviness, duration, and cessation (*N* = 462,690, mean age 56.7 years, SD = 8.0 years) (25).

Alcohol consumption is defined as ever regularly drinking alcohol or not (24). The mean age at the time of alcohol consumption assessment was 29.0 years (SD = 1.8 years). Alcohol intake frequency comprised of 462,346 participants aged 40–69 years was assessed through the IEU OpenGWAS project (ukb-b-5779). For coffee, GWAS selected includes coffee intake defined as drinking coffee regularly or not from MRC-IEU (ukb-b-5237). The GWAS of tea intake defined as drinking tea regularly from MRC-IEU (ukb-b-6066) comprised 447,485 participants aged 40–69 years.

For leisure lifestyles, participants were asked on a typical day how many hours they spend watching TV, using the computer (excluding using a computer at work), and driving (15). The mean age at the time of assessment was 57.4 years (SD = 8.0 years).

For the main outcome, OSA, the GWAS summary statistics were from the FinnGen database (26) comprised of 217,955 individuals with 16,761 OSA patients and 201,194 controls. The diagnosis of OSA was based on ICD codes (ICD-10: G47.3; ICD-9: 3472A), which were obtained from the Finnish National Hospital Discharge Registry and the Causes of Death Registry. This diagnosis is based on subjective symptoms, clinical examination, and sleep registration applying AHI ≥5 events·h^−1^ or respiratory event index ≥5 events·h^−1^. The median age at the first event of OSA is 56.46 years (Table 1, Supplementary Table 1).

**Table 1:** Summary of genome-wide association study (GWAS) datasets included in our study.

**Traits**	**Year**	**Sample size**	**Sources**	**Population**
**Smoking behaviors**				
Ever smoked regularly	2019	1,232,091	GSCAN	European
Age at initiation of regular smoking	2019	341,427	GSCAN	European
Cigarettes per day	2019	337,334	GSCAN	European
Smoking cessation	2019	547,219	GSCAN	European
Lifetime smoking index	2019	462,690	UK Biobank	European
**Drink intake**				
Alcohol consumption	2019	941,280	GSCAN	European
Alcohol intake frequency	2018	462,346	MRC-IEU	European
Coffee intake	2018	428,860	MRC-IEU	European
Tea intake	2018	447,485	MRC-IEU	European
**Leisure sedentary behaviors**				
Television watching	2020	422,218	UK Biobank	European
Driving	2020	422,218	UK Biobank	European
Computer use	2020	422,218	UK Biobank	European
**Sleep apnea**				
OSA	2021	217,955	FinnGen	European

OSA, obstructive sleep apnea.

### 2.3 Genetic instrument

We selected single nucleotide polymorphisms (SNPs) associated with genome-wide significance levels (*p* < 5 × 10^−8^) and chain disequilibrium *r*^2^ ≤ 0.001 within the distance of 10,000 kb as a strict cutoff of linkage disequilibrium, ensuring independence before being used as primary genetic instruments. Every paired combination was obtained for further analysis after coordinating with responsive outcomes. The details of genetic instruments are shown in Supplementary Tables 2–4.

### 2.4 Statistical analyses

Generally, we performed a two-sample MR analysis after harmonizing the SNPs in the data source with the same allele. First, the inverse-variance weighted (IVW) method is considered the primary method to estimate the causal effect of modifiable lifestyle factors on OSA. Then, to verify the consistency of our results and analyze sensitivity, MR-Egger (27, 28), weighted median (29), MR-PRESSO (30), and MR RAPS (arXiv:1801.09652, assessed on 2024/01/09) were applied. MR-Egger (27, 28) does not assume that all genetic instruments are valid and hence were used in scenarios where pleiotropy may be present. The primary assumption of MR-Egger is the Instrument Strength Independent of Direct Effect (InSIDE) assumption. This posits that the strength of the genetic instruments should not be associated with the potential direct effects of the exposure on the outcome. The method incorporates an intercept term into the analysis, allowing for the estimation of average pleiotropic effects. Mendelian Randomization Pleiotropy RESidual Sum and Outlier (30) (MR-PRESSO) addresses challenges in MR analyses by detecting and correcting for horizontal pleiotropy. It identifies and removes outliers, genetic variants exhibiting potential pleiotropy, and conducts a global test to assess and correct for horizontal pleiotropy. MR-PRESSO enhances the robustness of causal inference by providing an adjusted estimate that accounts for the influence of pleiotropic effects. Furthermore, the p-value of the primary method is applied to adjust for instances of false-positive results.

Cochran's *Q* heterogeneity test was applied to evaluate the degree of heterogeneity, and *p* < 0.05 was considered a high level of heterogeneity. We identified and removed the outliers by radial MR (31) and then repeated the above analysis. The instrument strength in our study was assessed using *F*-statistics. *F*-statistics >10 was set as the threshold. The statistical analyses were conducted based on R version 4.1.0 and TwoSampleMR version 0.5.6.

### 2.5 Ethics statement

This study is conducted based on publicly available data from UKBiobank, GSCAN and FinnGen studies. Ethical approval was granted for each of UKBiobank and FinnGen, informed consent was obtained from all participants before participation. Each GWAS study had received approval from a relevant institutional review board from their country, patient personal information in the databases is unidentifiable.

## 3 Results

### 3.1 Effects of smoking behaviors on OSA risk

**Figure 2:**
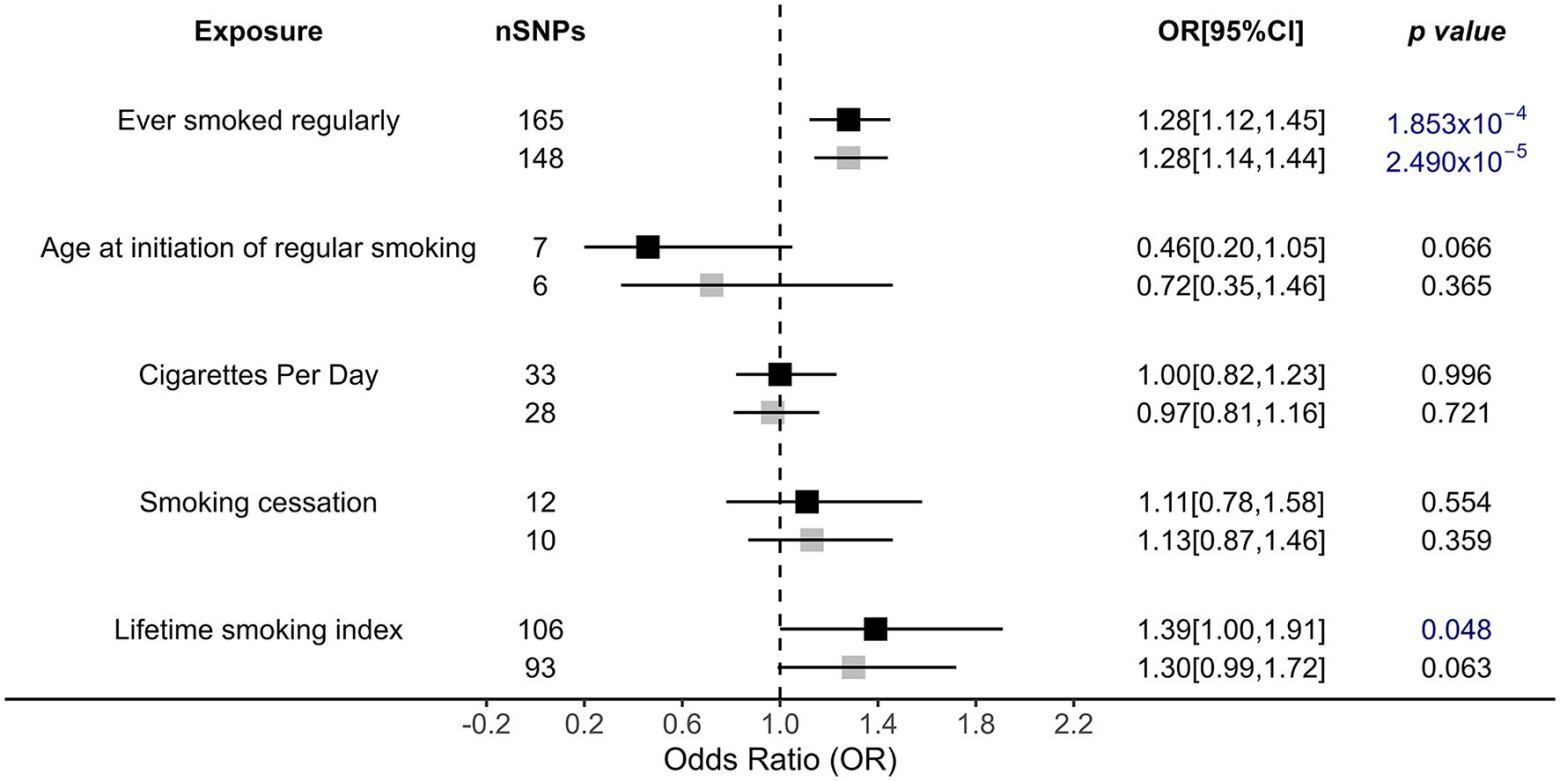
Causal impacts of smoking behaviors on OSA obtained from IVW analysis. Black illustrates the primary analysis with fulfilled SNPs as IVs. Gray illustrates the results with outlier-removed variants as IVs. The vertical dotted line delineates an OR of 1. OR, odds ratio; 95% CI, 95% confidence interval; OSA, obstructive sleep apnea; IVs, instrumental variables.

There was a causal effect of regular smoking history on OSA risk by the IVW primary analysis, and this result was robust after a more rigorous Bonferroni test [OR = 1.28, 95% CI = (1.12, 1.45), *p* = 1.853 × 10^−4^ < 0.05/12], i.e., those who ever smoked regularly had a 1.28 times higher risk of developing OSA than those who never smoked (Figure 2). Similar associations were also observed and were stable in MR-Egger [OR = 1.66, 95% CI = (1.00, 2.76), *p* = 0.05], weighted median [OR = 1.29, 95% CI = (1.09, 1.53), *p* = 2.66 × 10^−3^], and MR RAPS [OR = 1.28, 95% CI = (1.15, 1.42), *p* = 9.20 × 10^−6^; Figure 2, Supplementary Table 5]. In addition, the lifetime smoking index, a composite of smoking initiation, heaviness, duration, and cessation also had a causal role in OSA risk [OR = 1.39, 95% CI = (1.00, 1.91), *p* = 0.048], with a one-unit lifetime smoking index increasing 39% OSA risk (Figure 2). However, the results were not significant for the other applied methods (Figure 2, Supplementary Table 5). Additionally, age at initiation of regular smoking, cigarettes per day, and smoking cessation showed no causal association with OSA.

### 3.2 Causal impact of drink behaviors on OSA risk

**Figure 3:**
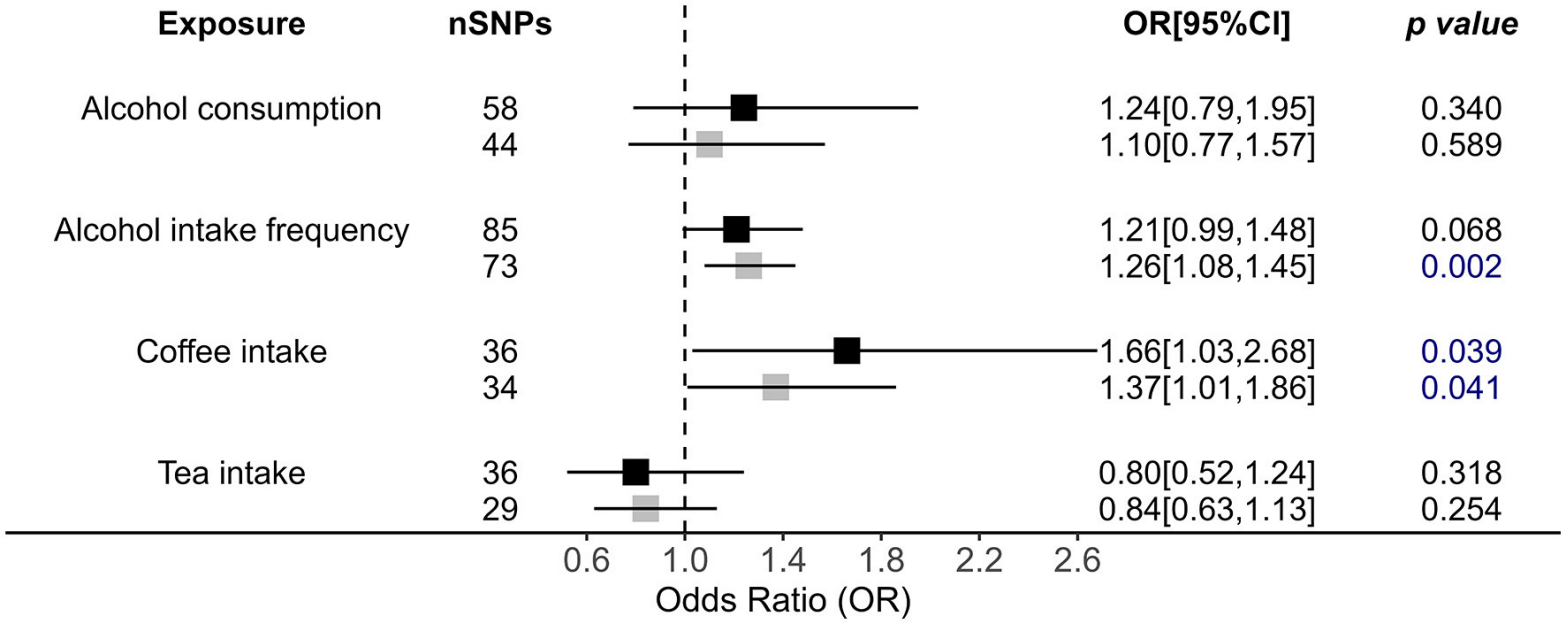
Causal impacts of alcohol, coffee, and tea intake behaviors on OSA obtained from IVW analysis. Black illustrates the primary analysis with fulfilled SNPs as IVs. Gray illustrates the results with outlier-removed variants as IVs. The vertical dotted line delineates an OR of 1. OR, odds ratio; 95% CI, 95% confidence interval; OSA, obstructive sleep apnea; IVs, instrumental variables.

For drinking alcohol behaviors, we only observed that alcohol intake frequency [outliers removed IVW OR = 1.26, 95% (1.08, 1.45), *p* = 0.002] had a causal effect on increasing OSA risks (Figure 3). MR RAPS also supports this conclusion [outliers removed OR = 1.28, 95% CI = (1.11, 1.47), *p* = 5.56 × 10^−4^]. Although the significant relationship was confirmed by the weighted median and MR RAPS methods, MR-Egger showed a reverse direction, calling for further validation of this association (Figure 3, Supplementary Table 5).

In addition, we also observed causality for coffee intake on increasing OSA risk by primary method [OR = 1.66, 95% CI = (1.03, 2.68), *p* = 0.039; Figure 3]; i.e., those who regularly drink coffee have a 1.66 times higher risk of developing OSA. MR RAPS also confirmed the role of coffee intake in OSA [OR = 1.59, 95% CI = (1.19, 2.12), *p* = 1.62 × 10^−3^]. However, after performing a more rigorous *p-*value adjustment test, this causality disappeared by IVW analysis. Additionally, tea intake showed no causal impact on OSA (Figure 3, Supplementary Table 5).

### 3.3 The effects of leisure behaviors showed no causal role in OSA

**Figure 4:**
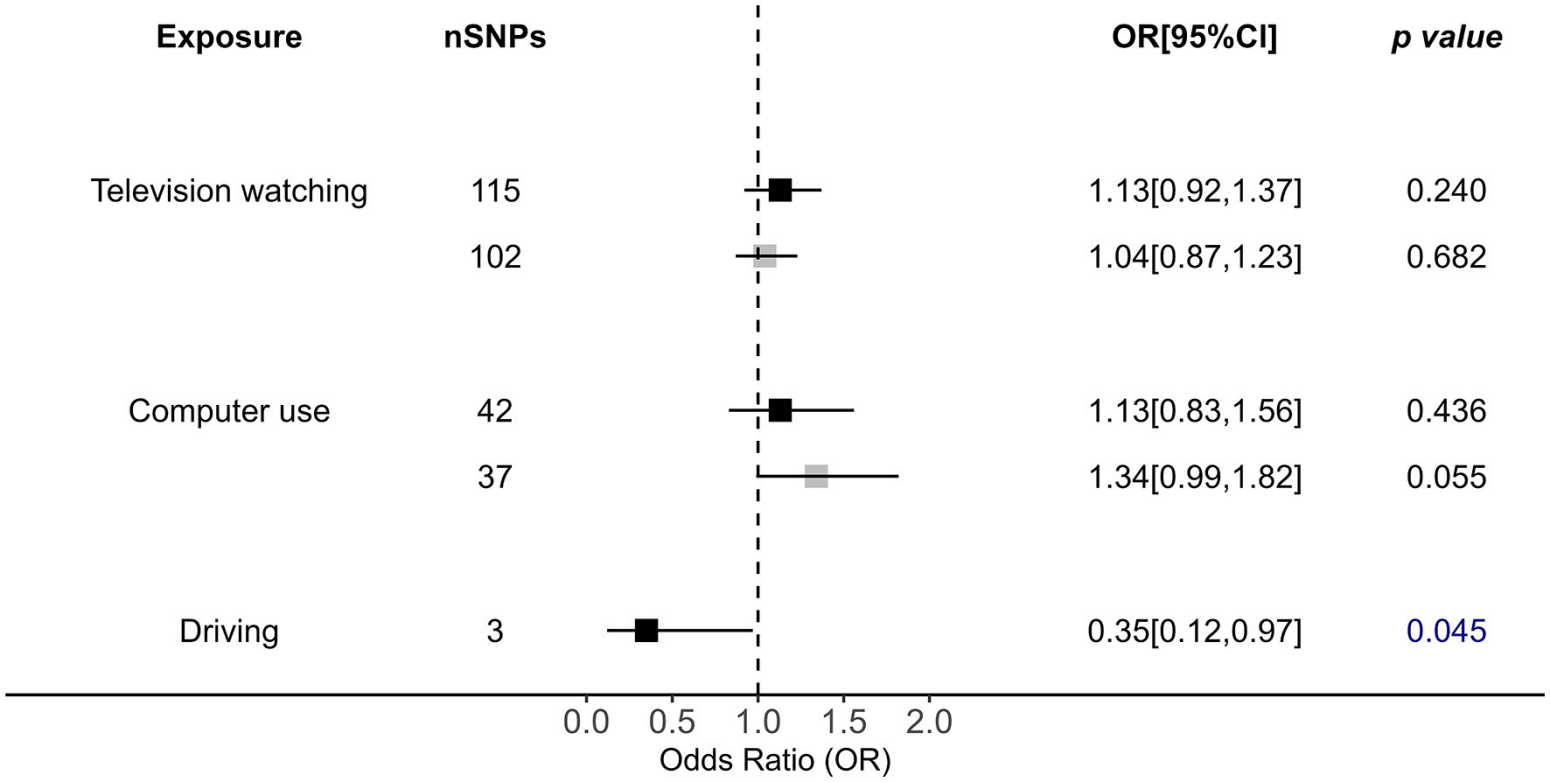
Causal impacts of leisure sedentary behaviors (including television watching, computer use, and driving) behaviors on OSA obtained from IVW analysis. Black illustrates the primary analysis with fulfilled SNPs as IVs. Gray illustrates the results with outlier-removed variants as IVs. The vertical dotted line delineates an OR of 1. OR, odds ratio; 95% CI, 95% confidence interval; OSA, obstructive sleep apnea; IVs, instrumental variables.

In terms of leisure behaviors, TV watching (outliers removed OR = 1.04, *p* = 0.682), and computer use (outliers removed OR = 1.34, *p* = 0.055) showed no causal impact on OSA (*P*_IVW_ > 0.05; Figure 4). Driving time is also causally associated with OSA risk by IVW analysis (OR = 0.35, *p* = 0.045), but this causality is unstable after more rigorous *p*-value adjustment (Figure 4). However, its *p*-value was close to the significant cutoff, and this result was not confirmed in any other applied approaches, suggesting that the association was unstable (Figure 4, Supplementary Table 5).

### 3.4 Sensitivity analysis

All possible causal associations identified by the IVW method showed no significant heterogeneity or horizontal pleiotropy (Cochran's *Q* test *p* > 0.05, MR-Egger Intercept *p* > 0.05; Supplementary Table 6). Leave-one-out analysis confirmed the results that were not driven by any single SNP.

## 4 Discussion

This study comprehensively evaluated the casual association between common modifiable lifestyles (including smoking, alcohol, coffee, tea consumption, TV watching, computer use, and driving) and OSA via an MR approach. Our results showed that ever regularly smoking showed a robust causal role in increasing OSA risks. Frequent alcohol intake, lifetime smoking index, and coffee intake are risk factors for OSA. Although the clinical association between these lifestyles and OSA has been widely reported by observational studies (8, 9, 32), our research confirmed the causal role of these lifestyles in OSA risk. Hopefully, our results will provide some insights for evidence-based precaution and clinical care for OSA.

First, we found that both ever-regular smoking and the lifetime smoking index play causal roles in increasing the risk of OSA. Although the clinical association between cigarette smoking and OSA has been widely reported by observational studies, our research first confirmed the causal role of smoking in OSA pathogenesis. Although this causal relationship is still not well understood, there are certain theories. One possible explanation for smoking's effect on sleep apnea is nicotine's impairment of upper airway neuromuscular protective reflexes (33). Additionally, smoking could change sleep structure into a higher proportion of light sleep (namely, the N1 and N2 periods) and correspondingly a lower proportion of slow-wave sleep (the N3 period), while sleep apnea episodes are also more likely to occur in the N1 period. Meanwhile, smoking could also contribute to a hyperarousal state (34), which could also increase the risk for sleep apnea episodes. Aside from enhancing OSA itself, smoking could also increase the chances of common complications, especially cardiovascular diseases and hypertension (35).

However, neither smoking cessation nor cigarettes per day showed any effects on OSA risk. This is inconsistent with previous clinical research reporting that increased pack-years could increase OSAS severity (32). Although smoking cessation should theoretically enhance sleep health by withdrawing from this harmful lifestyle, both our results and several previous clinical studies reported otherwise. In a large epidemiologic study, former smokers were found not to have a significantly increased prevalence of sleep-disordered breathing, as compared with non-smokers (36). To our knowledge, there have been no previous clinical trials on the impact of smoking cessation on OSA patients. Nicotine is a highly addictive substance, while during smoking cessation, increased insomnia and irritability, even mimicking OSA symptoms, were observed, especially in the first 1–2 days of smoking cessation (33). However, after fighting through this tough acute withdrawal phase, smoking cessation should still benefit sleep health in the long term. One previous clinical study observed better sleep quality in former smokers than in current smokers (37).

In addition, we observed a probable causal association between frequent alcohol intake and increased OSA risks. This finding is consistent with clinical research reporting that alcohol consumption may increase OSA risks (8, 9) and severity (38). We also offer some possible explanations for this causal role of frequent alcohol use in increasing OSA. Alcohol has a depressant effect on respiratory centers in the central nervous system, which controls the tone of pharyngeal muscles (39). Therefore, alcohol may increase the susceptibility to pharyngeal closure and subsequent upper airway obstruction during sleep. Additionally, there may be a potential dose–effect relationship with moderate drinking decreases the risk of OSA (40), whereas problematic drinking increases the OSA risk. For instance, the Mediterranean diet encourages moderate use of wine, which has been widely recognized to protect against risks of OSA and cardiovascular diseases (41, 42).

For common stimulant drinks in daily life (coffee, tea), we found that coffee intake has a probable causal role in OSA risk. For leisure behaviors, although we observed no robust causal impact on OSA, computer use showed a trend for increasing OSA risk after outlier removal (OR = 1.34, *p* = 0.055). Only a few clinical studies have reported that coffee and tea may increase OSA risks, while there has not been a previous MR analysis focusing on their causal association. Our findings, while highlighting the causal role of coffee consumption on OSA risks, also provide practical lifestyle advice for OSA patients. Daytime sleepiness or daytime function impairments are common symptoms of OSA (43), while coffee, as the most common stimulant drink (44), may seem to be the perfect fix. However, considering that coffee may also increase OSA risk, it may be better in other alternative ways (i.e., moderate outdoor exercise) to cope with daytime symptoms of OSA.

The definition of ever smoked regularly, age at initiation of regular smoking, cigarettes per day, and the lifetime smoking index encompasses various dimensions of smoking behavior. However, nuances in self-reported smoking history and potential recall biases should be acknowledged. Similarly, for alcohol consumption, the categorization into ever regularly drinking or not simplifies a complex behavior, potentially oversimplifying the spectrum of alcohol consumption patterns. Careful consideration of these definitional nuances is crucial to interpreting the associations between smoking, alcohol, and OSA accurately. Regarding the duration of measurement, our observations on smoking, alcohol consumption, and lifestyle span multiple years; however, the dynamic nature of individual behaviors might not be fully captured over extended periods. Despite our efforts in mitigating confounding factor BMI, the possibility of unaccounted confounders remains. Therefore, further studies should consider the dose effect and develop more specific health advice for drinking alcohol in OSA patients.

Additionally, in our study, there is none of the sample overlap between the exposure and outcome datasets, as they are derived from distinct cohorts. The genetic variant–exposure associations are independently evaluated in each dataset, ensuring that the assessments are specific to their respective sources. Furthermore, the exposure and outcome samples are sourced from different cohorts, emphasizing the robustness of our study design in mitigating confounding factors and bolstering the credibility of the observed associations.

Our study still has certain limitations. First, the definition of OSA from the FinnGen database in our study relies on ICD codes. In addition, it is acknowledged that without individual screening or a sleep study in the control group, subtle variations in OSA status could exist. In addition, while efforts were made to comprehensively define exact behavior, the retrospective nature of data collection may introduce recall bias, impacting the accuracy of self-reported information. Additionally, there is considerable heterogeneity in this study, which may reduce the statistical significance of our findings. Meanwhile, we extensively explored reverse causality in various facets of our analysis and observed OSA exhibited a weak causal impact on increased coffee intake [beta = 0.09, 95% CI = (4.98 × 10^−3^, 0.17), *p* = 0.04] and alcohol intake frequency [outliers removed beta = 0.08, 95% CI = (0.01, 0.14), *p* = 0.02]. The potential for bias in our study demands careful consideration, with attention to both direction and magnitude. Last but not least, the GWAS used in this study mainly included populations from Europe, which may make our findings less applicable in other regions. In addition, it is essential to acknowledge that the validity of MR assumptions, including relevance, no confounding, and no pleiotropy. While we have endeavored to adhere to established protocols and methodologies, the potential for deviations in MR assumptions remains an intrinsic challenge. These limitations collectively emphasize the need for a careful interpretation of our findings and underscore the importance of ongoing methodological advancements in MR research.

In conclusion, ever regularly smoking showed a robust causal role in OSA risks. Frequent alcohol intake, lifetime smoking index, and coffee intake are probable causalities for OSA. These lifestyles should be discouraged as precautions from developing OSA. More advanced studies are still needed to further authenticate these associations.

## Data availability statement

The original contributions presented in the study are included in the article/Supplementary material, further inquiries can be directed to the corresponding authors.

## Author contributions

KL: Data curation, Methodology, Writing – original draft, Formal analysis, Resources, Visualization. CZ: Data curation, Formal analysis, Methodology, Resources, Writing – original draft, Conceptualization. JW: Writing – review & editing, Data curation, Methodology, Software. JL: Conceptualization, Data curation, Methodology, Software, Writing – original draft, Writing – review & editing. ZC: Writing – review & editing, Data curation, Methodology, Software. MH: Writing – review & editing, Data curation, Methodology, Software. BL: Writing – review & editing, Data curation, Methodology, Software. XS: Writing – review & editing, Data curation, Methodology, Software. YZ: Writing – original draft, Data curation, Methodology, Software. MY: Conceptualization, Data curation, Funding acquisition, Investigation, Methodology, Software, Supervision, Writing – original draft, Writing – review & editing.
