# Supplementary material for: Effects of common lifestyle factors on obstructive sleep apnea: precautions in daily life based on causal inferences
Source: Front Public Health. 2024 Mar 5;12:1264082. doi: 10.3389/fpubh.2024.1264082 (PMC10948455; doi:10.3389/fpubh.2024.1264082)
Supplement: Supplementary file 1 [file Table_1.pdf]

Supplementary 1: supplemental tables

Supplemental Table 1. Detailed information on GWAS datasets in this study.

Supplemental Table 2. Characteristic of genetic instruments associated with smoking behaviors, alcohol, coffee and tea intake, leisure sedentary behaviors.

Supplemental Table 3. Characteristic of genetic instruments associated with OSA.

Supplemental Table 4. Summary of mean F-statistic for smoking behaviors, alcohol, coffee and tea intake, leisure sedentary behaviors, OSA and selected genetic variants.

Supplemental Table 5. Two-Sample Mendelian Randomization results investigating the cause relationships between smoking behaviors, alcohol, coffee and tea intake, leisure sedentary behaviors and OSA.

Supplemental Table 6. The heterogeneity and sensitivity results of smoking behaviors, alcohol, coffee and tea intake, leisure sedentary behaviors and OSA.

Supplemental Table 1. Detailed information on GWAS datasets in this study.

Traits
**Age of Initiation of Regular Smoking (AgeSmk)**
Definition: Age at which an individual started smoking cigarettes regularly.
Exclusion: Does not include information about pipes, cigars, chew, or other non-cigarette forms of tobacco use.
Measurement: Assessed through questions such as the age at which regular smoking began or by combining the duration of smoking with the current age.
**Cigarettes per Day (CigDay)**
Definition: Average number of cigarettes smoked per day, including both self-rolled and manufactured cigarettes.
Exclusion: Does not include information about pipes, cigars, chew, or other non-cigarette forms of tobacco use.
Binning: For studies with quantitative measures, responses were binned into categories (e.g., 1-5, 6-15).
Measurement: Often assessed through a single question about daily cigarette consumption.
**Smoking Cessation (SmkCes)**
Phenotype: Binary, with current smokers coded as "2," former smokers coded as "1," and never smokers coded as missing.
Exclusion: Does not include information about pipes, cigars, chew, or other non-cigarette forms of tobacco use.
Measurement: Usually determined through a combination of questions about current smoking status and past regular smoking.
**Smoking Initiation (Smklnit)**
Phenotype: Binary, with participants reporting ever being a regular smoker coded as "2" and those reporting never being a regular smoker coded as "1."
Exclusion: Does not include information about pipes, cigars, or other non-cigarette forms of tobacco use.
Measurement: Assessed through various questions related to smoking history, such as smoking over 100 cigarettes or smoking regularly.
**Drinks per Week (DrnkWk)**
Definition: Average number of drinks consumed per week, across all types of alcohol.
Binning: If recorded in ranges, the midpoint was used.
Measurement: Assessed through questions about alcohol consumption in the past week or on average over the past year.
Transformation: Left-anchored at 1 and log-transformed to prevent outliers from disproportionately affecting analyses.
**Lifetime smoking index**
Creation: Generated using data from the UK Biobank, combining smoking measures into an index with a simulated half-life constant.
Participants: 462,690 individuals of European ancestry from the UK Biobank.
Half-life Constant: Simulated at 18, capturing the decreasing effect of smoking on health outcomes over time.
**Leisure Sedentary Behavior**
Participants: 422,218 individuals of European ancestry from the UK Biobank.
Exclusions: 81 excluded, 1337 failed genetic quality control, and 77,469 were of non-European ancestry.
Sedentary Time: Mean daily reported leisure television watching was 2.8 h, leisure computer use was 1.0 h, and driving was 0.9 h.

Supplemental Table 2. Characteristic of genetic instruments associated with smoking behaviors, alcohol, coffee and tea intake, leisure sedentary behaviors.

Exposure	SNP	F-statistic
Ever smoked regularly	rs1004787	123.00
Ever smoked regularly	rs1022376	31.90
Ever smoked regularly	rs10233018	93.20
Ever smoked regularly	rs10279261	51.80
Ever smoked regularly	rs1030015	31.40
Ever smoked regularly	rs10490159	43.70
Ever smoked regularly	rs1050847	33.40
Ever smoked regularly	rs10698713	35.60
Ever smoked regularly	rs10853981	29.80
Ever smoked regularly	rs10858334	37.00
Ever smoked regularly	rs10885480	43.70
Ever smoked regularly	rs10945141	39.30
Ever smoked regularly	rs10953957	30.30
Ever smoked regularly	rs10966092	50.60
Ever smoked regularly	rs11057005	37.50
Ever smoked regularly	rs1106363	42.00
Ever smoked regularly	rs11076962	41.50
Ever smoked regularly	rs1108130	59.00
Ever smoked regularly	rs1109480	40.60
Ever smoked regularly	rs1116690	31.30
Ever smoked regularly	rs1126757	30.80
Ever smoked regularly	rs112725451	58.90
Ever smoked regularly	rs113230003	41.70
Ever smoked regularly	rs1139897	63.30
Ever smoked regularly	rs1150668	51.20
Ever smoked regularly	rs11594623	83.20
Ever smoked regularly	rs11611651	35.90
Ever smoked regularly	rs11642231	34.90
Ever smoked regularly	rs11713899	30.60
Ever smoked regularly	rs117657830	35.10
Ever smoked regularly	rs11783093	182.00
Ever smoked regularly	rs11791671	30.00
Ever smoked regularly	rs118202	127.00
Ever smoked regularly	rs1187820	30.90
Ever smoked regularly	rs12195240	77.90
Ever smoked regularly	rs12474587	88.60
Ever smoked regularly	rs12517438	36.10
Ever smoked regularly	rs12633090	48.60
Ever smoked regularly	rs12714017	34.80
Ever smoked regularly	rs12878369	45.40
Ever smoked regularly	rs12918191	44.10
Ever smoked regularly	rs1291821	32.20
Ever smoked regularly	rs13007361	31.20
Ever smoked regularly	rs13110073	89.40

	Ever smoked regularly	rs13261666	61.50
	Ever smoked regularly	rs13392222	40.50
	Ever smoked regularly	rs13437771	59.30
	Ever smoked regularly	rs1373178	61.60
	Ever smoked regularly	rs1381287	49.70
	Ever smoked regularly	rs1381775	30.80
	Ever smoked regularly	rs1385108	39.20
	Ever smoked regularly	rs13906	36.00
	Ever smoked regularly	rs1435672	30.20
	Ever smoked regularly	rs1445649	64.80
	Ever smoked regularly	rs1449012	36.20
	Ever smoked regularly	rs1549979	87.40
	Ever smoked regularly	rs160631	36.10
	Ever smoked regularly	rs16826827	33.00
	Ever smoked regularly	rs1714521	39.60
	Ever smoked regularly	rs17197663	31.40
	Ever smoked regularly	rs17229285	36.90
	Ever smoked regularly	rs1733760	33.60
	Ever smoked regularly	rs1772572	38.40
	Ever smoked regularly	rs1901477	136.00
	Ever smoked regularly	rs2010921	40.10
	Ever smoked regularly	rs2063976	55.90
	Ever smoked regularly	rs2155646	211.00
	Ever smoked regularly	rs2173019	71.40
	Ever smoked regularly	rs221988	32.20
	Ever smoked regularly	rs2279829	31.50
	Ever smoked regularly	rs2289791	36.00
	Ever smoked regularly	rs2319545	42.20
	Ever smoked regularly	rs2378662	35.40
	Ever smoked regularly	rs2587507	33.10
	Ever smoked regularly	rs2710634	48.50
	Ever smoked regularly	rs2796793	32.00
	Ever smoked regularly	rs281296	86.20
	Ever smoked regularly	rs28441558	41.40
	Ever smoked regularly	rs290601	32.60
	Ever smoked regularly	rs2925128	39.30
	Ever smoked regularly	rs2939756	37.90
	Ever smoked regularly	rs3218116	46.20
	Ever smoked regularly	rs329124	40.50
	Ever smoked regularly	rs34342129	31.40
	Ever smoked regularly	rs34399632	41.10
	Ever smoked regularly	rs34553878	37.00
	Ever smoked regularly	rs35375873	44.00
	Ever smoked regularly	rs359247	69.00
	Ever smoked regularly	rs3764351	30.20
	Ever smoked regularly	rs3810496	36.50
	Ever smoked regularly	rs3811038	45.40
	Ever smoked regularly	rs3847244	53.50

Ever smoked regularly	rs3934797	41.60
Ever smoked regularly	rs4044321	72.40
Ever smoked regularly	rs4264267	33.60
Ever smoked regularly	rs4310804	37.90
Ever smoked regularly	rs4476253	38.40
Ever smoked regularly	rs4674993	56.80
Ever smoked regularly	rs4727189	30.70
Ever smoked regularly	rs4752018	38.90
Ever smoked regularly	rs4790874	46.70
Ever smoked regularly	rs4818005	59.70
Ever smoked regularly	rs4822102	39.80
Ever smoked regularly	rs4837631	35.90
Ever smoked regularly	rs4877285	44.90
Ever smoked regularly	rs540860	47.40
Ever smoked regularly	rs58400863	56.80
Ever smoked regularly	rs59537158	52.40
Ever smoked regularly	rs6050446	51.10
Ever smoked regularly	rs6073075	31.10
Ever smoked regularly	rs61533748	44.30
Ever smoked regularly	rs61886926	46.90
Ever smoked regularly	rs61959481	42.30
Ever smoked regularly	rs62007780	37.90
Ever smoked regularly	rs62193862	31.50
Ever smoked regularly	rs62246017	35.20
Ever smoked regularly	rs62340589	30.00
Ever smoked regularly	rs6265	80.60
Ever smoked regularly	rs6437769	30.30
Ever smoked regularly	rs6438436	56.60
Ever smoked regularly	rs644740	30.30
Ever smoked regularly	rs6497840	63.10
Ever smoked regularly	rs6568832	40.70
Ever smoked regularly	rs67050670	44.70
Ever smoked regularly	rs6731872	88.40
Ever smoked regularly	rs6750529	46.50
Ever smoked regularly	rs6756212	176.00
Ever smoked regularly	rs67777803	53.10
Ever smoked regularly	rs6874731	36.10
Ever smoked regularly	rs6890961	53.90
Ever smoked regularly	rs6936160	52.50
Ever smoked regularly	rs6986430	63.10
Ever smoked regularly	rs7024924	31.60
Ever smoked regularly	rs7072776	61.00
Ever smoked regularly	rs7134009	30.00
Ever smoked regularly	rs71367544	42.10
Ever smoked regularly	rs71592686	52.70
Ever smoked regularly	rs71602617	31.40
Ever smoked regularly	rs72780746	58.50
Ever smoked regularly	rs73008357	31.10

Ever smoked regularly	rs748832	42.60
Ever smoked regularly	rs75919030	53.00
Ever smoked regularly	rs7631379	43.60
Ever smoked regularly	rs76608582	34.20
Ever smoked regularly	rs7696257	33.60
Ever smoked regularly	rs77283305	30.20
Ever smoked regularly	rs7743165	57.10
Ever smoked regularly	rs7809303	62.00
Ever smoked regularly	rs7836565	30.00
Ever smoked regularly	rs7929518	40.00
Ever smoked regularly	rs7943721	39.30
Ever smoked regularly	rs7969559	36.50
Ever smoked regularly	rs8005334	39.40
Ever smoked regularly	rs8027457	36.10
Ever smoked regularly	rs8096225	31.00
Ever smoked regularly	rs9288999	36.50
Ever smoked regularly	rs9302604	53.00
Ever smoked regularly	rs9323328	31.00
Ever smoked regularly	rs9423279	48.70
Ever smoked regularly	rs9540731	48.40
Ever smoked regularly	rs9545155	39.70
Ever smoked regularly	rs9627272	35.60
Ever smoked regularly	rs9826984	30.20
Ever smoked regularly	rs9922607	48.40
Ever smoked regularly	rs9941217	48.40
Age at initiation of regular smoking	rs11780471	41.90
Age at initiation of regular smoking	rs11915747	63.50
Age at initiation of regular smoking	rs13136239	33.70
Age at initiation of regular smoking	rs2471711	32.50
Age at initiation of regular smoking	rs624833	35.70
Age at initiation of regular smoking	rs72853300	31.80
Age at initiation of regular smoking	rs7682598	35.90
Cigarettes Per Day	rs10204824	50.30
Cigarettes Per Day	rs1024323	33.10
Cigarettes Per Day	rs10454798	32.00
Cigarettes Per Day	rs10519203	1310.00
Cigarettes Per Day	rs10742683	29.80
Cigarettes Per Day	rs113001570	37.30
Cigarettes Per Day	rs11846838	34.20
Cigarettes Per Day	rs11940255	40.30
Cigarettes Per Day	rs12924872	30.00
Cigarettes Per Day	rs1592485	41.60
Cigarettes Per Day	rs182317	36.80
Cigarettes Per Day	rs2084533	38.20
Cigarettes Per Day	rs215600	89.00
Cigarettes Per Day	rs2273500	112.00
Cigarettes Per Day	rs258321	41.00
Cigarettes Per Day	rs28813180	40.50

Cigarettes Per Day	rs3025383	101.00
Cigarettes Per Day	rs4236926	142.00
Cigarettes Per Day	rs4485470	38.00
Cigarettes Per Day	rs56113850	447.00
Cigarettes Per Day	rs6078373	41.90
Cigarettes Per Day	rs62447179	32.90
Cigarettes Per Day	rs632811	40.80
Cigarettes Per Day	rs699165	33.30
Cigarettes Per Day	rs7125588	47.20
Cigarettes Per Day	rs7281463	30.60
Cigarettes Per Day	rs73229090	46.10
Cigarettes Per Day	rs7431710	50.80
Cigarettes Per Day	rs7599488	33.10
Cigarettes Per Day	rs7766641	39.70
Cigarettes Per Day	rs78408772	29.90
Cigarettes Per Day	rs790564	41.40
Cigarettes Per Day	rs7951365	45.50
Smoking cessation	rs12203592	32.50
Smoking cessation	rs1565735	50.00
Smoking cessation	rs1611124	34.10
Smoking cessation	rs518425	49.80
Smoking cessation	rs56113850	214.00
Smoking cessation	rs591143	37.10
Smoking cessation	rs6011779	101.00
Smoking cessation	rs707968	30.90
Smoking cessation	rs7109376	41.60
Smoking cessation	rs7617480	49.80
Smoking cessation	rs7778443	32.80
Smoking cessation	rs9607805	45.70
Lifetime smoking index	rs10052591	35.89
Lifetime smoking index	rs10226228	63.04
Lifetime smoking index	rs10282292	38.37
Lifetime smoking index	rs1050847	32.18
Lifetime smoking index	rs10879871	43.17
Lifetime smoking index	rs112282219	43.71
Lifetime smoking index	rs11255908	40.22
Lifetime smoking index	rs113382419	162.66
Lifetime smoking index	rs11783093	68.62
Lifetime smoking index	rs11861214	31.48
Lifetime smoking index	rs11948770	38.71
Lifetime smoking index	rs12202536	35.29
Lifetime smoking index	rs1221148	42.42
Lifetime smoking index	rs1246265	34.51
Lifetime smoking index	rs12481282	33.32
Lifetime smoking index	rs12623702	46.84
Lifetime smoking index	rs12708665	34.87
Lifetime smoking index	rs12831617	31.63
Lifetime smoking index	rs12967855	30.64

	Lifetime smoking index	rs13009008	34.33
	Lifetime smoking index	rs13016665	36.14
	Lifetime smoking index	rs13153393	40.00
	Lifetime smoking index	rs13296519	46.75
	Lifetime smoking index	rs136233	31.67
	Lifetime smoking index	rs147412694	35.27
	Lifetime smoking index	rs17309874	50.91
	Lifetime smoking index	rs17553262	34.07
	Lifetime smoking index	rs17576594	49.81
	Lifetime smoking index	rs1922018	48.70
	Lifetime smoking index	rs202645	34.69
	Lifetime smoking index	rs2062882	32.62
	Lifetime smoking index	rs2080870	29.76
	Lifetime smoking index	rs2254710	30.43
	Lifetime smoking index	rs245774	33.41
	Lifetime smoking index	rs2675638	36.84
	Lifetime smoking index	rs2838834	38.22
	Lifetime smoking index	rs28485305	30.96
	Lifetime smoking index	rs2867112	61.34
	Lifetime smoking index	rs2890772	94.82
	Lifetime smoking index	rs2894808	34.86
	Lifetime smoking index	rs317021	41.72
	Lifetime smoking index	rs326341	45.91
	Lifetime smoking index	rs329120	47.24
	Lifetime smoking index	rs34866095	32.42
	Lifetime smoking index	rs348809	32.37
	Lifetime smoking index	rs35169606	36.95
	Lifetime smoking index	rs35175834	93.27
	Lifetime smoking index	rs35343344	33.10
	Lifetime smoking index	rs359243	37.42
	Lifetime smoking index	rs369230	36.18
	Lifetime smoking index	rs3742365	58.10
	Lifetime smoking index	rs3811038	37.56
	Lifetime smoking index	rs3896224	46.10
	Lifetime smoking index	rs421983	39.49
	Lifetime smoking index	rs4391802	45.61
	Lifetime smoking index	rs4473348	42.68
	Lifetime smoking index	rs4543592	38.89
	Lifetime smoking index	rs4571506	32.03
	Lifetime smoking index	rs4671357	46.15
	Lifetime smoking index	rs4731925	30.96
	Lifetime smoking index	rs4814873	35.23
	Lifetime smoking index	rs4957528	34.55
	Lifetime smoking index	rs57611503	30.15
	Lifetime smoking index	rs6011779	117.47
	Lifetime smoking index	rs60952428	30.73
	Lifetime smoking index	rs6119897	61.93
	Lifetime smoking index	rs61796681	30.05

Lifetime smoking index	rs62098013	34.58
Lifetime smoking index	rs62135536	37.76
Lifetime smoking index	rs62155874	65.73
Lifetime smoking index	rs62175972	31.79
Lifetime smoking index	rs624833	38.14
Lifetime smoking index	rs6598539	34.41
Lifetime smoking index	rs6741228	31.94
Lifetime smoking index	rs67596067	37.04
Lifetime smoking index	rs6778080	50.36
Lifetime smoking index	rs6779302	36.98
Lifetime smoking index	rs6935954	46.71
Lifetime smoking index	rs6957896	29.90
Lifetime smoking index	rs6962772	33.33
Lifetime smoking index	rs7039819	38.65
Lifetime smoking index	rs7077678	35.47
Lifetime smoking index	rs71367545	36.69
Lifetime smoking index	rs7155595	35.53
Lifetime smoking index	rs71627581	36.35
Lifetime smoking index	rs72674867	30.26
Lifetime smoking index	rs72678864	45.35
Lifetime smoking index	rs7297175	33.65
Lifetime smoking index	rs732083	32.12
Lifetime smoking index	rs73220544	32.00
Lifetime smoking index	rs7333559	39.58
Lifetime smoking index	rs74086911	31.39
Lifetime smoking index	rs7569203	51.44
Lifetime smoking index	rs75742406	36.84
Lifetime smoking index	rs76608582	39.54
Lifetime smoking index	rs7766610	49.27
Lifetime smoking index	rs7807019	56.16
Lifetime smoking index	rs8042134	50.34
Lifetime smoking index	rs8042849	172.80
Lifetime smoking index	rs860326	35.35
Lifetime smoking index	rs8614	40.69
Lifetime smoking index	rs889398	42.73
Lifetime smoking index	rs9842947	35.09
Lifetime smoking index	rs986391	60.01
Lifetime smoking index	rs9904288	30.62
Lifetime smoking index	rs9919670	115.06
Alcohol consumption	rs10236149	37.00
Alcohol consumption	rs10438820	31.70
Alcohol consumption	rs10506274	38.40
Alcohol consumption	rs10750025	43.20
Alcohol consumption	rs10978550	42.50
Alcohol consumption	rs11030084	31.80
Alcohol consumption	rs1123285	33.20
Alcohol consumption	rs11692435	44.50
Alcohol consumption	rs11940694	305.00

Alcohol consumption	rs1217091	42.50
Alcohol consumption	rs1229984	1520.00
Alcohol consumption	rs1260326	197.00
Alcohol consumption	rs12655091	32.40
Alcohol consumption	rs12795042	30.60
Alcohol consumption	rs12907323	32.90
Alcohol consumption	rs13024996	51.90
Alcohol consumption	rs13032049	39.70
Alcohol consumption	rs13094887	42.10
Alcohol consumption	rs13107325	95.40
Alcohol consumption	rs13250583	29.80
Alcohol consumption	rs13383034	92.60
Alcohol consumption	rs17177078	54.90
Alcohol consumption	rs17665139	31.90
Alcohol consumption	rs2011092	33.40
Alcohol consumption	rs2165670	95.30
Alcohol consumption	rs2472297	39.60
Alcohol consumption	rs281379	88.60
Alcohol consumption	rs2854334	37.90
Alcohol consumption	rs28601761	38.00
Alcohol consumption	rs28929474	45.80
Alcohol consumption	rs35034355	30.80
Alcohol consumption	rs3748034	31.80
Alcohol consumption	rs3803800	41.00
Alcohol consumption	rs3809162	37.00
Alcohol consumption	rs4092465	30.00
Alcohol consumption	rs4548913	30.60
Alcohol consumption	rs4690727	44.60
Alcohol consumption	rs4815364	32.80
Alcohol consumption	rs4916723	45.30
Alcohol consumption	rs4938230	41.10
Alcohol consumption	rs500321	34.20
Alcohol consumption	rs55872084	33.70
Alcohol consumption	rs55932213	32.90
Alcohol consumption	rs56030824	55.10
Alcohol consumption	rs56337305	40.90
Alcohol consumption	rs62044525	41.80
Alcohol consumption	rs62250685	91.60
Alcohol consumption	rs6460047	41.90
Alcohol consumption	rs6787172	30.00
Alcohol consumption	rs682011	31.30
Alcohol consumption	rs7074871	31.60
Alcohol consumption	rs72859280	34.40
Alcohol consumption	rs77165542	42.90
Alcohol consumption	rs7950166	41.80
Alcohol consumption	rs828867	35.80
Alcohol consumption	rs9607814	30.00
Alcohol consumption	rs9838144	30.90

Alcohol consumption	rs9950000	37.40
Alcohol intake frequency	rs10188314	42.48
Alcohol intake frequency	rs10792669	32.87
Alcohol intake frequency	rs11039429	60.16
Alcohol intake frequency	rs11223617	44.68
Alcohol intake frequency	rs11700855	32.42
Alcohol intake frequency	rs11750777	30.25
Alcohol intake frequency	rs11787216	58.19
Alcohol intake frequency	rs11940694	196.80
Alcohol intake frequency	rs12153855	35.60
Alcohol intake frequency	rs1228589	35.67
Alcohol intake frequency	rs1229984	811.86
Alcohol intake frequency	rs12312693	33.60
Alcohol intake frequency	rs13102973	38.72
Alcohol intake frequency	rs13135092	63.55
Alcohol intake frequency	rs13178443	30.27
Alcohol intake frequency	rs13390019	43.46
Alcohol intake frequency	rs1421085	41.78
Alcohol intake frequency	rs1515591	34.22
Alcohol intake frequency	rs1666658	33.62
Alcohol intake frequency	rs17662759	30.46
Alcohol intake frequency	rs17690703	53.26
Alcohol intake frequency	rs186347	34.62
Alcohol intake frequency	rs1893659	92.26
Alcohol intake frequency	rs1937522	31.06
Alcohol intake frequency	rs1991083	47.24
Alcohol intake frequency	rs2159935	37.68
Alcohol intake frequency	rs2160935	36.67
Alcohol intake frequency	rs2411453	128.85
Alcohol intake frequency	rs2535911	35.38
Alcohol intake frequency	rs2622167	38.84
Alcohol intake frequency	rs262240	32.15
Alcohol intake frequency	rs2717063	43.61
Alcohol intake frequency	rs28622224	30.56
Alcohol intake frequency	rs28768122	33.96
Alcohol intake frequency	rs2924321	40.93
Alcohol intake frequency	rs2977454	31.78
Alcohol intake frequency	rs34440851	29.87
Alcohol intake frequency	rs34473884	33.78
Alcohol intake frequency	rs34631026	30.78
Alcohol intake frequency	rs34811474	31.55
Alcohol intake frequency	rs35105141	72.79
Alcohol intake frequency	rs362307	55.70
Alcohol intake frequency	rs4241258	32.40
Alcohol intake frequency	rs4242715	32.98
Alcohol intake frequency	rs461599	39.85
Alcohol intake frequency	rs4726481	49.22
Alcohol intake frequency	rs473098	51.03

Alcohol intake frequency	rs489062	29.74
Alcohol intake frequency	rs4916723	59.70
Alcohol intake frequency	rs4968391	35.67
Alcohol intake frequency	rs5022348	32.22
Alcohol intake frequency	rs550942	31.54
Alcohol intake frequency	rs56194430	30.65
Alcohol intake frequency	rs58905411	74.85
Alcohol intake frequency	rs6030200	35.65
Alcohol intake frequency	rs61873510	38.04
Alcohol intake frequency	rs62305780	91.74
Alcohol intake frequency	rs62339673	33.64
Alcohol intake frequency	rs62466318	45.62
Alcohol intake frequency	rs650558	34.94
Alcohol intake frequency	rs6727281	38.50
Alcohol intake frequency	rs71651683	30.35
Alcohol intake frequency	rs72769229	30.46
Alcohol intake frequency	rs72787062	47.22
Alcohol intake frequency	rs728538	31.70
Alcohol intake frequency	rs7298932	30.27
Alcohol intake frequency	rs7302200	33.18
Alcohol intake frequency	rs73050128	40.41
Alcohol intake frequency	rs7330939	39.25
Alcohol intake frequency	rs74679146	31.03
Alcohol intake frequency	rs76082653	48.21
Alcohol intake frequency	rs7610856	60.42
Alcohol intake frequency	rs780094	269.71
Alcohol intake frequency	rs80292319	36.74
Alcohol intake frequency	rs8043563	45.31
Alcohol intake frequency	rs838145	51.63
Alcohol intake frequency	rs8614	39.85
Alcohol intake frequency	rs9349379	39.40
Alcohol intake frequency	rs9372625	66.90
Alcohol intake frequency	rs9403297	36.16
Alcohol intake frequency	rs9648478	30.99
Alcohol intake frequency	rs9814516	49.87
Alcohol intake frequency	rs9829192	30.81
Alcohol intake frequency	rs9906502	36.05
Alcohol intake frequency	rs9912298	32.89
Coffee intake	rs1057868	124.90
Coffee intake	rs117810762	33.78
Coffee intake	rs117968677	31.65
Coffee intake	rs12514566	44.65
Coffee intake	rs12989746	30.82
Coffee intake	rs13054099	34.46
Coffee intake	rs13163336	45.74
Coffee intake	rs1338549	33.96
Coffee intake	rs13387939	59.93
Coffee intake	rs1421085	127.16

Coffee intake	rs1527961	31.81
Coffee intake	rs17842490	44.01
Coffee intake	rs1942965	30.24
Coffee intake	rs2189234	36.17
Coffee intake	rs2465037	38.77
Coffee intake	rs2472297	646.73
Coffee intake	rs2597805	31.49
Coffee intake	rs34060476	60.45
Coffee intake	rs4410790	545.51
Coffee intake	rs442355	36.10
Coffee intake	rs476828	83.75
Coffee intake	rs56113850	60.13
Coffee intake	rs57918684	33.14
Coffee intake	rs6062682	40.02
Coffee intake	rs6063085	38.90
Coffee intake	rs61928609	45.85
Coffee intake	rs62064918	30.10
Coffee intake	rs630194	44.68
Coffee intake	rs6469262	31.58
Coffee intake	rs73075167	43.19
Coffee intake	rs75347775	30.93
Coffee intake	rs780093	64.37
Coffee intake	rs7811609	30.14
Coffee intake	rs78267637	34.70
Coffee intake	rs8056750	36.78
Coffee intake	rs9398171	37.21
Tea intake	rs10741694	46.78
Tea intake	rs10752269	36.88
Tea intake	rs10764990	31.59
Tea intake	rs1156588	35.24
Tea intake	rs12591786	39.27
Tea intake	rs13282783	33.29
Tea intake	rs132904	42.30
Tea intake	rs141071726	35.75
Tea intake	rs1453548	35.17
Tea intake	rs1481012	61.15
Tea intake	rs149805207	32.68
Tea intake	rs17245213	31.52
Tea intake	rs17576658	30.12
Tea intake	rs17685	95.36
Tea intake	rs2273447	43.99
Tea intake	rs2279844	30.15
Tea intake	rs2351187	31.96
Tea intake	rs2472297	493.65
Tea intake	rs2478875	70.30
Tea intake	rs2645929	30.42
Tea intake	rs34619	30.02
Tea intake	rs4410790	341.27

Tea intake	rs4808193	45.24
Tea intake	rs4817505	48.01
Tea intake	rs56348300	33.80
Tea intake	rs57462170	31.62
Tea intake	rs57631352	31.87
Tea intake	rs6829	30.28
Tea intake	rs713598	38.59
Tea intake	rs72797284	51.56
Tea intake	rs7757102	30.62
Tea intake	rs9302428	30.95
Tea intake	rs9624470	136.84
Tea intake	rs9648476	32.72
Tea intake	rs977474	58.18
Tea intake	rs9937354	43.23
Television watching	rs10041724	43.65
Television watching	rs10054327	61.99
Television watching	rs10145592	45.20
Television watching	rs10189857	88.11
Television watching	rs1022785	33.51
Television watching	rs10234444	30.77
Television watching	rs1031423	49.73
Television watching	rs10771746	35.57
Television watching	rs10772643	50.31
Television watching	rs10876864	37.30
Television watching	rs10932837	36.76
Television watching	rs11130793	34.17
Television watching	rs11201422	30.46
Television watching	rs11218575	49.41
Television watching	rs11245482	35.49
Television watching	rs114600294	37.79
Television watching	rs1156541	30.87
Television watching	rs116175338	32.17
Television watching	rs11657730	32.68
Television watching	rs11689199	70.16
Television watching	rs11714337	43.29
Television watching	rs11763734	33.22
Television watching	rs12105701	34.03
Television watching	rs12272012	31.64
Television watching	rs12289262	31.51
Television watching	rs1243182	63.04
Television watching	rs12476388	31.40
Television watching	rs12491503	38.48
Television watching	rs12541615	39.15
Television watching	rs12554512	89.09
Television watching	rs13029509	72.15
Television watching	rs13107325	50.07
Television watching	rs138256022	30.75
Television watching	rs1421334	62.90

Television watching	rs1563908	53.91
Television watching	rs17207890	47.10
Television watching	rs17512836	38.02
Television watching	rs17727474	35.14
Television watching	rs17789218	54.72
Television watching	rs2034768	46.62
Television watching	rs2045147	33.88
Television watching	rs2073869	40.97
Television watching	rs2164744	32.75
Television watching	rs2173650	34.37
Television watching	rs2447098	47.08
Television watching	rs2460	38.69
Television watching	rs2616830	57.81
Television watching	rs262890	62.13
Television watching	rs2717559	32.09
Television watching	rs2787374	32.42
Television watching	rs28457808	31.99
Television watching	rs303753	39.85
Television watching	rs34864022	36.66
Television watching	rs35574015	30.69
Television watching	rs374722	65.61
Television watching	rs3754970	47.77
Television watching	rs3796386	144.19
Television watching	rs405797	35.29
Television watching	rs42210	33.44
Television watching	rs4334769	31.36
Television watching	rs4382592	33.63
Television watching	rs4435081	31.22
Television watching	rs4523073	38.68
Television watching	rs457335	34.09
Television watching	rs4577309	54.49
Television watching	rs4675246	30.80
Television watching	rs4775373	34.32
Television watching	rs4810315	30.90
Television watching	rs4973576	37.25
Television watching	rs55700114	35.88
Television watching	rs56103247	39.23
Television watching	rs56291505	31.29
Television watching	rs56858768	39.43
Television watching	rs57585211	34.47
Television watching	rs6131281	53.16
Television watching	rs6141814	36.80
Television watching	rs62151809	52.05
Television watching	rs62379379	37.81
Television watching	rs62490165	32.75
Television watching	rs6472942	36.21
Television watching	rs6551301	36.47
Television watching	rs6721975	40.77

Television watching	rs6754968	39.24
Television watching	rs6797840	54.36
Television watching	rs6825241	61.29
Television watching	rs6850494	41.58
Television watching	rs6905544	73.84
Television watching	rs6973656	37.28
Television watching	rs6996198	30.41
Television watching	rs707919	31.77
Television watching	rs7089973	35.65
Television watching	rs7184800	51.24
Television watching	rs7189927	43.95
Television watching	rs7248205	39.46
Television watching	rs72781699	48.66
Television watching	rs72828890	34.21
Television watching	rs72834698	53.39
Television watching	rs749671	48.92
Television watching	rs7564130	44.34
Television watching	rs7693082	40.84
Television watching	rs7693703	35.37
Television watching	rs7700107	45.05
Television watching	rs7865801	31.73
Television watching	rs7991062	60.14
Television watching	rs801733	55.98
Television watching	rs8043253	30.45
Television watching	rs8756	38.55
Television watching	rs9471333	36.49
Television watching	rs9563168	43.47
Television watching	rs9718104	78.20
Television watching	rs973734	32.11
Television watching	rs9834970	35.02
Television watching	rs9867121	48.19
Television watching	rs9902312	43.36
Television watching	rs9964724	57.53
Computer use	rs10208088	34.45
Computer use	rs10754920	33.63
Computer use	rs113851275	35.06
Computer use	rs11708955	43.52
Computer use	rs11749912	38.71
Computer use	rs12706626	32.22
Computer use	rs12874776	42.26
Computer use	rs13262595	50.45
Computer use	rs136553	44.69
Computer use	rs1448355	41.29
Computer use	rs1469249	32.94
Computer use	rs150254595	30.34
Computer use	rs162894	32.71
Computer use	rs166835	34.95
Computer use	rs198262	30.20

Computer use	rs2068625	43.64
Computer use	rs206965	33.02
Computer use	rs2220599	49.12
Computer use	rs2345941	43.64
Computer use	rs2734849	37.72
Computer use	rs35933007	33.00
Computer use	rs3730399	30.53
Computer use	rs3944151	30.66
Computer use	rs4702	30.24
Computer use	rs4977839	79.56
Computer use	rs55772938	39.14
Computer use	rs56158102	30.67
Computer use	rs6129084	37.46
Computer use	rs6498759	30.90
Computer use	rs66643547	44.05
Computer use	rs6744254	52.06
Computer use	rs6774533	36.95
Computer use	rs6857629	36.12
Computer use	rs6935828	30.00
Computer use	rs7020477	32.46
Computer use	rs7209653	42.01
Computer use	rs7288455	34.65
Computer use	rs73578186	40.18
Computer use	rs78082503	32.66
Computer use	rs784256	42.77
Computer use	rs9372625	65.53
Computer use	rs9477970	32.82
Driving	rs10186876	37.96
Driving	rs4765541	34.13
Driving	rs6012558	40.90

Supplemental Table 3. Characteristic of genetic instruments associated with OSA.

SNP	F-statistic
rs10507084	44.30821634
rs10928560	30.87982695
rs142006783	29.73084009
rs4837016	31.899904
rs9937053	66.5856

Supplemental Table 4. Summary of mean F-statistic for smoking behaviors, alcohol, coffee and tea intake, leisure sedentary behaviors, OSA and selected genetic variants.

Exposure	nSNP	F-statistic(mean)
Ever smoked regularly	165	48.61
Age at initiation of regular smoking	7	39.29
Cigarettes Per Day	33	98.10
Smoking cessation	12	59.94
Lifetime smoking index	106	44.33
Alcohol consumption	58	75.22
Alcohol intake frequency	85	55.77
Coffee intake	36	76.49
Tea intake	36	63.96
Television watching	115	43.61
Computer use	42	38.79
Driving	3	37.67
OSA	5	40.68

Supplemental Table 5. Two-Sample Mendelian Randomization results investigating the cause relationships between smoking behaviors, alcohol, coffee and tea intake, leisure sedentary behaviors and OSA.

	Method	nSNPs	OR[95%CI]/ BETA[95%CI]*	*p value*
**Ever smoked regularly→OSA**				
Original	MR Egger	165	1.66[1.00,2.76]	0.05
removed outliers		148	1.48[0.93,2.35]	0.10
Original	Weighted median	165	1.29[1.09,1.53]	**$2.66x10^{-3}$**
removed outliers		148	1.28[1.08,1.52]	**$3.74x10^{-3}$**
Original	MR RAPS	-	1.28[1.15,1.42]	**$9.20x10^{-6}$**
removed outliers		-	1.29[1.15,1.45]	**$1.44x10^{-5}$**
Original	MR PRESSO	165	1.28[1.12,1.45]	**$2.56x10^{-4}$**
**Age at initiation of regular smoking→OSA**				
Original	MR Egger	7	0.81[0.01,52.49]	0.92
removed outliers		6	2.16[0.11,40.68]	0.63
Original	Weighted median	7	0.61[0.24,1.58]	0.31
removed outliers		6	0.80[0.32,2.00]	0.64
Original	MR RAPS	-	0.46[0.26,0.79]	**$5.19x10^{-3}$**
removed outliers		-	0.65[0.35,1.19]	0.17
Original	MR PRESSO	7	0.46[0.20,1.05]	0.12
**Cigarettes Per Day→OSA**				
Original	MR Egger	33	0.87[0.62,1.23]	0.45
removed outliers		28	0.89[0.66,1.21]	0.47
Original	Weighted median	33	0.88[0.68,1.13]	0.32
removed outliers		28	0.88[0.68,1.13]	0.31
Original	MR RAPS	-	1.01[0.85,1.21]	0.88
removed outliers		-	0.98[0.82,1.18]	0.84
Original	MR PRESSO	33	1.00[0.82,1.23]	1.00
**Lifetime smoking index→OSA**				
Original	MR Egger	106	2.26[0.61,8.34]	0.23
removed outliers		93	1.55[0.52,4.65]	0.44
Original	Weighted median	106	1.22[0.81,1.85]	0.34
removed outliers		93	1.19[0.78,1.80]	0.42
Original	MR RAPS	-	1.42[1.10,1.85]	**0.01**
removed outliers		-	1.27[0.96,1.68]	0.09
Original	MR PRESSO	106	1.39[1.00,1.91]	0.05
**Smoking cessation→OSA**				
Original	MR Egger	12	0.80[0.27,2.40]	0.70
removed outliers		10	0.70[0.33,1.47]	0.37
Original	Weighted median	12	1.04[0.75,1.46]	0.80
removed outliers		10	1.05[0.76,1.45]	0.76
Original	MR RAPS	-	1.12[0.88,1.43]	0.37
removed outliers		-	1.13[0.87,1.47]	0.35
Original	MR PRESSO	12	1.11[0.78,1.58]	0.57
removed outliers		10	1.13[0.93,1.37]	0.25
**Alcohol consumption→OSA**				
Original	MR Egger	58	2.52[0.86,7.36]	0.10

removed outliers		44	1.23[0.46,3.33]	0.68
Original	Weighted median	58	1.49[0.89,2.48]	0.13
removed outliers		44	1.30[0.75,2.26]	0.34
Original	MR RAPS	-	1.30[0.96,1.77]	0.09
removed outliers		-	1.17[0.82,1.67]	0.40
Original	MR PRESSO	58	1.24[0.79,1.95]	0.34
**Alcohol intake frequency→OSA**				
Original	MR Egger	85	0.58[0.32,1.05]	0.08
removed outliers		73	0.64[0.39,1.04]	0.07
Original	Weighted median	85	1.09[0.88,1.35]	0.43
removed outliers		73	1.25[1.00,1.56]	**$4.97x10^{-2}$**
Original	MR RAPS	-	1.24[1.10,1.41]	**$7.61x10^{-4}$**
removed outliers		-	1.28[1.11,1.47]	**$5.56x10^{-4}$**
Original	MR PRESSO	85	1.21[0.99,1.48]	0.07
removed outliers		82	1.17[0.99,1.38]	0.07
**OSA→Alcohol intake frequency**				
original	IVW	5	0.11[0.03,0.18]*	**$6.19x10^{-3}$**
removed outliers		3	0.08[0.01,0.14]*	**0.02**
original	MR Egger	5	0.35[$-5.03x10^{-3}$,0.70]*	0.15
removed outliers		3	0.09[-0.25,0.43]*	0.70
original	Weighted median	5	0.09[0.02,0.16]*	**0.02**
removed outliers		3	0.07[$-9.84x10^{-3}$,0.14]*	0.09
original	MR RAPS	-	0.12[0.07,0.16]*	**$8.84x10^{-8}$**
removed outliers		-	0.08[$8.03x10^{-3}$,0.15]*	**0.03**
Original	MR PRESSO	5	0.11[0.03,0.18]*	0.05
**Coffee intake→OSA**				
Original	MR Egger	36	1.70[0.65,4.50]	0.29
removed outliers		34	1.27[0.70,2.31]	0.44
Original	Weighted median	36	1.35[0.87,2.07]	0.18
removed outliers		34	1.34[0.86,2.08]	0.19
Original	MR RAPS	-	1.59[1.19,2.12]	**$1.62x10^{-3}$**
removed outliers		-	1.35[1.00,1.83]	**$4.97x10^{-2}$**
Original	MR PRESSO	36	1.66[1.03,2.68]	**$4.67x10^{-2}$**
removed outliers		35	1.30[0.93,1.81]	0.13
**OSA→Coffee intake**				
Original	IVW	5	0.09[$4.98x10^{-3}$,0.17]*	**0.04**
	MR Egger	5	0.20[-0.25,0.65]*	0.45
	Weighted median	5	0.02[-0.01,0.06]*	0.22
	MR RAPS	-	0.10[0.08,0.13]*	**$2.22x10^{-16}$**
removed outliers	-	1	0.03[-0.02,0.08]*	0.27
Original	MR PRESSO	5	0.09[$4.98x10^{-3}$,0.17]*	0.11
removed outliers		2	0.02[-0.01,0.05]*	0.46
**Tea intake→OSA**				
Original	MR Egger	36	1.60[0.65,3.94]	0.31

removed outliers		29	0.86[0.42,1.78]	0.69
Original	Weighted median	36	1.02[0.67,1.56]	0.92
removed outliers		29	0.95[0.62,1.46]	0.82
Original	MR RAPS	-	0.80[0.63,1.02]	0.07
removed outliers		-	0.85[0.63,1.15]	0.29
Original	MR PRESSO	36	0.80[0.52,1.24]	0.32
removed outliers		34	0.78[0.57,1.06]	0.12
**OSA→Tea intake**				
Original	IVW	5	-0.05[-0.13,0.04]*	0.26
removed outliers		2	-0.01[-0.06,0.04]*	0.70
Original	MR Egger	5	-0.13[-0.61,0.34]*	0.61
Original	Weighted median	5	-0.01[-0.06,0.04]*	0.72
Original	MR RAPS	-	-0.06[-0.08,-0.03]*	**$3.56x10^{-5}$**
removed outliers		-	-0.01[-0.07,0.04]*	0.71
Original	MR PRESSO	5	-0.05[-0.13,0.04]*	0.33
removed outliers		3	0.00[-0.03,0.04]*	0.82
**Television watching→OSA**				
Original	MR Egger	115	1.18[0.46,3.00]	0.73
removed outliers		102	1.17[0.53,2.57]	0.70
Original	Weighted median	115	1.08[0.85,1.38]	0.52
removed outliers		102	1.07[0.84,1.36]	0.57
Original	MR RAPS	-	1.10[0.94,1.28]	0.25
removed outliers		-	1.05[0.89,1.24]	0.57
Original	MR PRESSO	115	1.13[0.92,1.37]	0.24
removed outliers		114	1.10[0.90,1.33]	0.35
**OSA→Television watching**				
Original	IVW	5	0.01[-0.03,0.06]*	0.63
removed outliers		4	0.03[$-7.29x10^{-3}$,0.07]*	0.12
Original	MR Egger	5	0.14[-0.08,0.35]*	0.30
removed outliers		4	-0.02[-0.33,0.29]*	0.90
Original	Weighted median	5	0.01[-0.03,0.04]*	0.74
removed outliers		4	0.01[-0.03,0.05]*	0.67
Original	MR RAPS	-	0.01[-0.02,0.04]*	0.42
removed outliers		-	0.03[$-2.58x10^{-3}$,0.06]*	0.07
Original	MR PRESSO	5	0.01[-0.03,0.06]*	0.65
**Computer use→OSA**				
Original	MR Egger	42	1.68[0.21,13.05]	0.63
removed outliers		37	0.78[0.11,5.60]	0.80
Original	Weighted median	42	1.13[0.75,1.72]	0.55
removed outliers		37	1.22[0.81,1.85]	0.35
Original	MR RAPS	-	1.14[0.86,1.51]	0.37
removed outliers		-	1.35[0.99,1.85]	0.06
Original	MR PRESSO	42	1.13[0.83,1.56]	0.44
**OSA→Computer use**				
Original	IVW	5	0.00[-0.04,0.04]*	0.91
	MR Egger	5	-0.13[-0.30,0.05]*	0.25
	Weighted median	5	0.00[-0.04,0.04]*	0.92

	MR RAPS	-	0.00[-0.03,0.03]*	0.87
Original	MR PRESSO	5	0.00[-0.04,0.04]*	0.92
**Driving→OSA**				
Original	MR Egger	3	$1.06x10^{7}$[0.00,$1.90x10^{27}$]	0.62
	Weighted median	3	0.33[0.10,1.14]	0.08
	MR RAPS	-	0.34[0.12,1.02]	0.05
**OSA→Driving**				
Original	IVW	5	0.00[-0.05,0.05]*	0.94
removed outliers		4	-0.02[-0.05,0.01]*	0.26
Original	MR Egger	5	-0.08[-0.34,0.19]*	0.60
removed outliers		4	-0.07[-0.22,0.09]*	0.49
Original	Weighted median	5	-0.02[-0.05,0.02]*	0.41
removed outliers		4	-0.02[-0.06,0.02]*	0.32
Original	MR RAPS	-	0.00[-0.03,0.03]*	0.89
removed outliers		-	-0.02[-0.05,0.01]*	0.27
Original	MR PRESSO	5	0.00[-0.05,0.05]*	0.94

Abbreviations: nSNPs, single-nucleotide polymorphism; OR, odds ratio; MR RAPS, Mendelian Randomization-Robust Adjusted Profile Score.
p values in bold indicate they achieved the nominal significance ($p<0.05$).

Supplemental Table 6. The heterogeneity and sensitivity results of smoking behaviors, alcohol, coffee and tea intake, leisure sedentary behaviors and OSA.

Exposure→Outcome		nSNPs	MR Egger intercept		Cochran's heterogeneity test			
					IVW		Egger	
			Intercept value	*p value*	Q value	*p value*	Q value	*p value*
**Smoking behavior**								
Ever smoked regularly→OSA	Original	165	-5.37×10^{-3}	0.30	230.37	**4.88×10^{-4}**	228.86	**5.14×10^{-4}**
	removed outliers	148	-2.96×10^{-3}	0.53	118.37	0.96	117.96	0.96
Age at initiation of regular smoking→OSA	Original	7	-1.10×10^{-2}	0.79	11.08	0.09	10.92	**0.05**
	removed outliers	6	-2.07×10^{-2}	0.49	4.63	0.46	4.05	0.40
Cigarettes Per Day→OSA	Original	33	4.55×10^{-3}	0.35	43.4	0.09	42.17	0.09
	removed outliers	28	2.80×10^{-3}	0.52	11.57	1.00	11.15	1.00
Lifetime smoking index→OSA	Original	106	-5.24×10^{-3}	0.45	159.79	**4.58×10^{-4}**	158.92	**4.28×10^{-4}**
	removed outliers	93	-1.88×10^{-3}	0.75	78.15	0.85	78.05	0.83
Smoking cessation→OSA	Original	12	0.01	0.55	23.36	**0.02**	22.48	**0.01**
	removed outliers	10	0.02	0.21	5.01	0.83	3.18	0.92
**Alcohol intake**								
Alcohol consumption→OSA	Original	58	-9.53×10^{-3}	0.16	122.57	**1.06×10^{-6}**	118.34	**2.33×10^{-6}**
	removed outliers	44	-1.42×10^{-3}	0.81	33.82	0.84	33.76	0.81
Alcohol intake frequency→OSA	Original	85	1.87×10^{-2}	**0.01**	210.72	**7.22×10^{-13}**	195.31	**4.82×10^{-11}**
	removed outliers	73	1.69×10^{-2}	**0.01**	80.2	0.24	72.02	0.44
OSA→Alcohol intake frequency	Original	5	-2.24×10^{-2}	0.27	15.56	**3.67×10^{-3}**	9.61	**0.02**
	removed outliers	3	-1.27×10^{-3}	0.95	1.17	0.56	1.17	0.28
**Coffee and tea intake**								
Coffee intake→OSA	Original	36	-5.20×10^{-4}	0.95	94.98	**1.93×10^{-7}**	94.97	**1.13×10^{-7}**
	removed outliers	34	1.54×10^{-3}	0.77	33.48	0.44	33.38	0.40
OSA→Coffee intake	Original	5	-1.08×10^{-2}	0.64	60.97	**1.82×10^{-12}**	56.08	**4.05×10^{-12}**
Tea intake→OSA	Original	36	-1.53×10^{-2}	0.10	109.45	**1.36×10^{-9}**	100.92	**1.49×10^{-8}**
	removed outliers	29	-4.87×10^{-4}	0.94	17.1	0.95	17.09	0.93
OSA→Tea intake	Original	5	8.21×10^{-3}	0.73	36.79	**1.99×10^{-7}**	35.15	**1.13×10^{-7}**
**Leisure sedentary behaviors**								
Television watching→OSA	Original	115	-7.44×10^{-4}	0.92	173.52	**2.77×10^{-4}**	173.51	**2.21×10^{-4}**
	removed outliers	102	-1.94×10^{-3}	0.76	72.80	0.98	72.71	0.98

OSA→Television watching	Original	5	$-1.16x10^{-2}$	0.33	10.19	**0.04**	7.04	0.07
	removed outliers	4	$5.35x10^{-3}$	0.77	4.07	0.25	3.85	0.15
Computer use→OSA	Original	42	$-6.06x10^{-3}$	0.71	53.50	**$9.14x10^{-2}$**	53.31	**$7.76x10^{-2}$**
	removed outliers	37	$8.45x10^{-3}$	0.58	24.31	0.93	24.01	0.92
OSA→Computer use	Original	5	0.01	0.23	7.69	0.10	4.41	0.22
Driving→OSA	Original	3	-0.25	0.60	1.13	0.57	0.60	0.44
OSA→Driving	Original	5	$7.56x10^{-3}$	0.59	11.43	**0.02**	10.17	**0.02**
	removed outliers	4	$4.71x10^{-3}$	0.59	2.84	0.42	2.37	0.31

p values in bold indicate they achieved the nominal significance ($p<0.05$).
